# A Rare Case of Pseudocyesis in a Patient With Bipolar Disorder

**DOI:** 10.7759/cureus.10352

**Published:** 2020-09-10

**Authors:** Eduardo D Espiridion, Christina Fleckenstein, Patrick Boyle, Adeolu O Oladunjoye

**Affiliations:** 1 Psychiatry, Drexel University College of Medicine, Philadelphia, USA; 2 Psychiatry, West Virginia School of Osteopathic Medicine, Lewisburg, USA; 3 Psychiatry, West Virginia University School of Medicine, Martinsburg, USA; 4 Psychiatry, Philadelphia College of Osteopathic Medicine, Philadelphia, USA; 5 Psychiatry, Reading Hospital Tower Health, West Reading, USA; 6 Medicine, Drexel University College of Medicine, Philadelphia, USA; 7 Medicine, Philadelphia College of Osteopathic Medicine, Philadelphia, USA; 8 Medical Critical Care, Boston Children's Hospital; Harvard Medical School, Boston, USA

**Keywords:** pseudocyesis, delusion of pregnancy, bipolar disorders, psychosis, mania

## Abstract

Pseudocyesis is a rare condition in which a person has a false belief of being pregnant, accompanied by objective signs and symptoms of pregnancy, despite not being pregnant. Confirmation of pseudocyesis is achieved with a negative result of beta-human chorionic gonadotropin in the blood and/or urine and negative ultrasound finding. Most cases of pseudocyesis occur in the setting of major depressive disorder or psychotic disorder, with very few occurring during a manic episode of bipolar disorder. Hence, we present a 30-year-old woman with pseudocyesis in the setting of bipolar disorder, specifically within a current manic episode with features of psychosis.

The patient was found in the woods naked, with signs of psychosis. She described symptoms of increasing abdominal size, whitish discharge from her nipple, and feeling of fetal movement. The patient continued to believe she was pregnant due to her symptoms, despite negative pregnancy tests on multiple occasions. She has a history of bipolar disorder and post-traumatic stress disorder. Her examination showed an obese woman, with a non-distended abdomen and non-palpable uterus with no breast tenderness or enlargement. The patient was given olanzapine for her agitation and was subsequently stabilized with haloperidol and lorazepam. She was restarted on her home medications, including risperidone, oxcarbazepine, and topiramate. She was later committed involuntarily and transferred to a long-term psychiatry facility.

Pseudocyesis is a rare condition often associated with other psychiatric comorbidities. Our patient's presentation highlights one of the few cases ever formally documented in a developed country, as most of the cases reported are found in developing countries. More studies, including case series and systematic reviews, need to be done to better understand this rare condition and its other variants.

## Introduction

Pseudocyesis is a rare condition defined in the Diagnostic and Statistical Manual of Mental Disorders-5 (DSM-5), as when a person has a false belief of being pregnant, accompanied by objective signs and symptoms of pregnancy, which may include abdominal enlargement, oligomenorrhea/amenorrhea, subjective sensation of fetal movement, nausea, breast engorgement, and secretions, and labor pains at the expected date of delivery, despite not being pregnant [1]. Confirmation of pseudocyesis is achieved with negative pregnancy testing of blood and urine, or ultrasound [2].

Pseudocyesis and its other variants should be differentiated. The presence of physical symptoms of pregnancy is unique to pseudocyesis. On the other hand, the delusion of pregnancy is defined in the DSM-5 as when a person has a fixed belief of being pregnant but in the absence of physical signs and symptoms suggestive of pregnancy and negative confirmatory tests [3]. It is a true somatic delusion, characterized under schizophrenia and other psychotic disorders. Delusional pregnancy is common in women during the state of psychosis. Another differential diagnosis of pseudocyesis is factitious disorder imposed on self, defined in the DSM-5 as a category of somatic symptom disorder when a woman acknowledges being pregnant knowing that it is not true [4].

Pseudocyesis is rare and occurs more in developing countries [5]. As of 2016, fewer than six hundred cases had been formally documented worldwide [6]. Many of these cases occurred in the context of major depressive disorder [6-7] and psychotic disorders [8]. However, very few cases have been reported in patients with bipolar disorder [7]. Hence, we present a case of a woman with pseudocyesis in the setting of bipolar disorder, specifically within a current manic episode with features of psychosis.

## Case presentation

History of presenting complaint

A 30-year-old woman, high school graduate, who was single and lived with her mother and four children. The patient was brought to the emergency department (ED) by the police, after she was found in the woods, naked with signs of psychosis including persecutory delusion, auditory hallucination, and agitation. She claimed to be pregnant, validated by her subjective symptoms of increasing abdominal size, whitish discharge from her nipples, and feeling of fetal movement in her lower abdomen. The patient had a history of bipolar disorder and post-traumatic stress disorder (PTSD), which had been well-managed for years with medications and routine therapy.

Her last sexual encounter was seven months prior to presentation, which was followed by three months of amenorrhea. Five months prior to admission, she suspected she was pregnant and took several home pregnancy tests, which turned out negative. She consulted her primary care physician (PCP) who confirmed she was not pregnant. Despite evidence of not being pregnant, the patient had continued to believe that she was pregnant. She claimed to feel fetal movement in her lower abdomen and reported whitish discharge from her nipples, which resembled breast milk. She corroborated her story with the fact that her youngest child always told her she was pregnant with twins. She denied nausea, vomiting, breast enlargement, or skin changes.

One month prior to admission, the patient discontinued her psychiatric medications, which included risperidone, oxcarbazepine, and topiramate, worried that they might harm her pregnancy. This precipitated her manic symptoms with psychotic features, as the patient reported excessive energy and only two hours of sleep each night for the last two weeks. She also endorsed rapidity of speech, agitation, anxiety, delusions of being stalked, and auditory hallucinations of her deceased great-grandmother. A day prior to her previous hospitalization, a friend noticed her abnormal behavior and brought her to a psychiatric hospital, where she was given quetiapine and later discharged with recommended outpatient follow-up. Presumably after this encounter, her symptoms worsened and resulted in her current admission.

Examination

Physical examination showed an obese woman (body mass index: 35.45). Her abdominal examination showed a non-distended abdomen with a non-palpable uterus, supple breasts with no tenderness or enlargement. Mental status examination revealed a poorly groomed woman with messy hair and poor dentition. She was cooperative, calm, and made appropriate eye contact. Her speech was rapid but normally cadenced and appropriate in volume. Her affect was full range and euthymic. She was awake, alert, oriented to time, place, and person. However, she was not fully oriented to the events that precipitated her admission. Her thought process was illogical and tangential. Her judgment and insight were poor. For example, she justified her nakedness in the woods as a coping mechanism for anxiety related to delusions of being stalked.

Investigation and treatment

Five months prior to admission, when her urine pregnancy test was negative, her PCP ordered blood workup, which showed a negative blood pregnancy test, prolactin (PRL) level of 3.8 ng/mL (reference range: 3.3 - 26.7 ng/mL), and thyroid-stimulating hormone of 3.08 uIU/mL (reference range: 0.45 - 5.33 uIU/mL). The patient pulled out her intravenous (IV) line several times during her current admission, stating that she did not want the IV line anymore. At another time, she threatened to leave the hospital and threw a cellphone at her nurse. The patient was given olanzapine for her agitation and was subsequently stabilized with haloperidol and lorazepam. She was restarted on her home medications, including risperidone, oxcarbazepine, and topiramate for her bipolar disorder and propranolol for her migraine. She was later committed involuntarily and transferred to a long term psychiatry facility.

## Discussion

Patients with pseudocyesis may reveal oligomenorrhea or amenorrhea, increase in abdominal size, nipple discharge, the sensation of fetal movement [3,9], all of which our patient claimed to have experienced. However, our patient did not have other symptoms reported in the literature such as breast enlargement and increased pigmentation. The physiologic cause of pseudocyesis is not known. However, Brown et al. have proposed that mood disorders can lead to increased catecholamine levels, for example, dopamine [10], thereby downregulating gonadotropin and upregulating PRL and hence the galactorrhea with a decrease in follicular stimulating hormone (FSH) and luteinizing hormone (LH) leading to amenorrhea [11]. In our patient who claimed to experience galactorrhea, her hormone levels, including PRL, FSH, and LH, were normal. Beta-human chorionic gonadotropin, also taken on multiple occasions by urine and blood, was consistently negative. Endocrinological studies have also stated that certain hormonal states, including polycystic ovarian syndrome and hyperprolactinemia secondary to antipsychotic medications, can mimic pregnancy, potentially contributing to pseudocyesis [3]. However, our patient stopped her antipsychotic medications a month prior to admission and may have had amenorrhea due to her earlier use of antipsychotic medications.

Mood disorders, such as depression, have been implicated as a comorbidity in pseudocyesis [6], unlike our patient, whose mood disorder was mania with psychotic features. Pseudocyesis manifests differently depending on the psychiatric presentation. Those with manic symptoms present with grandiose ideas and thoughts while those with depression often present after postpartum psychosis or experience pseudo-hallucinations [5]. Pseudocyesis with manic symptoms typically feels the sense of uterine contractions and fetal movements similar to what our patient described in her history [12].

Pseudocyesis has been reported more in women from developing countries, with low socioeconomic status, limited access to health care, and significant stressors [3]. However, our patient does not fit any of these descriptions, which makes this case more unique. Tarrin et al. report that in developed countries, women have more access to diagnostic tests, including a pregnancy test and ultrasonography, leading to resolution of pseudocyesis before it is ever noticed; hence, the fewer cases of pseudocyesis in developed countries [3]. Pawlowski et al. also state that with the exposure of women to education in developed countries, emotional conflicts may be refined in expression rather than to think of pseudocyesis [13]. A study by Ouj reports that in some developing countries, importance is placed on having children so that any woman who wishes to get pregnant must confirm her womanhood by getting pregnant [14]. This makes them yield to societal pressures, which can precipitate pseudocyesis as a physiological defense to this intense stress.

The management of pseudocyesis is multidisciplinary, including psychiatrists, gynecologists, and psychologists [15]. The goal of treatment is to help patients perceive the meaning of their symptoms and to help resolve the associated stressors [15]. Psychotherapy such as supportive, cognitive, behavioral, and psychoanalytic psychotherapy has been found to be helpful [6]. These psychotherapies can be combined with medications like an antidepressant and antipsychotic medication depending on the associated comorbidities.

Our patient's case brings to focus the need to pay attention to rare conditions when ruling out differential diagnoses that are not common in certain locations. Also, early and adequate treatment of psychiatric symptoms will prevent worsening clinical conditions. For instance, if our patient was managed early and adequately during her previous hospitalization, her clinical condition may not have deteriorated.

## Conclusions

Pseudocyesis is a rare condition often associated with other psychiatric comorbidities. Our patient's presentation highlights one of the few cases ever formally documented in a developed country, as most of the cases reported are found in developing countries. To add to this is the unique presentation of bipolar disorder in our patient, which is a very rare occurrence in pseudocyesis. More studies, including case series and systematic reviews, need to be done to better understand this rare condition and its variants.
